# Metabolic Imaging in Prostate Cancer: Where We Are

**DOI:** 10.3389/fonc.2016.00225

**Published:** 2016-11-09

**Authors:** Claudia Testa, Cristian Pultrone, David Neil Manners, Riccardo Schiavina, Raffaele Lodi

**Affiliations:** ^1^Functional MR Unit, Department of Biomedical and Neuromotor Sciences, S. Orsola-Malpighi Hospital, University of Bologna, Bologna, Italy; ^2^Urologic Unit, Experimental, Diagnostic and Specialty Medicine, Department of Urology, S. Orsola-Malpighi Hospital, University of Bologna, Bologna, Italy

**Keywords:** metabolic imaging, prostate cancer, MRSI, PET/CT, multiparametric MRI

## Abstract

In recent years, the development of diagnostic methods based on metabolic imaging has been aimed at improving diagnosis of prostate cancer (PCa) and perhaps at improving therapy. Molecular imaging methods can detect specific biological processes that are different when detected within cancer cells relative to those taking place in surrounding normal tissues. Many methods are sensitive to tissue metabolism; among them, positron emission tomography (PET) and magnetic resonance spectroscopic imaging (MRSI) are widely used in clinical practice and clinical research. There is a rich literature that establishes the role of these metabolic imaging techniques as valid tools for the diagnosis, staging, and monitoring of PCa. Until recently, European guidelines for PCa detection still considered both MRSI/MRI and PET/CT to be under evaluation, even though they had demonstrated their value in the staging of high risk PCa, and in the restaging of patients presenting elevated prostatic-specific antigen levels following radical treatment of PCa, respectively. Very recently, advanced methods for metabolic imaging have been proposed in the literature: multiparametric MRI (mpMRI), hyperpolarized MRSI, PET/CT with the use of new tracers and finally PET/MRI. Their detection capabilities are currently under evaluation, as is the feasibility of using such techniques in clinical studies.

Prostate cancer (PCa) is the most common cancer in American men and in European elderly males (beyond 70 years of age). About one American man in seven and one European in eight will be diagnosed with PCa during his lifetime.

Although imaging has played a major role in PCa, many challenges still remain in the different phases of the disease: initial assessment during diagnosis, and re-assessment after radical treatment in cases of biochemical relapse and disease progression.

Currently, prostate biopsy remains the only procedure that provides a definitive diagnosis. The decision to perform a biopsy is based on information gathered from serum prostatic-specific antigen (PSA) level, digital rectal examination (DRE), ultra sound (US), and magnetic resonance (MR) imaging, along with family history of PCa. In the era of active, multimodal surveillance of PCa, clinicians are presented with challenge of improving the accuracy of biopsy during the diagnostic phase and detecting only the aggressive diseases that require treatment, to avoid overdiagnosis and overtreatment. Another significant challenge for imaging is PCa staging (1). Currently, lymph node staging is based on surgery. A greater accuracy of imaging for staging may avoid unnecessary lymph nodes dissections reducing related complications (2).

Last but not least, accurate imaging during restaging of PCa following radical treatment presents an unsolved challenge. Currently, when conventional imaging detects disease relapse, PSA and PSA kinetics are too high and patients are often outside the window of curability. A greater accuracy of metabolic imaging techniques should improve the efficacy of salvage treatments (3).

## Cell Metabolism in Prostate

Prostate gland metabolism, in both its healthy and malignant forms, can be considered a “model” that has improved our understanding of the mechanisms and factors occurring when normal cells transform themselves into malignant cells. The metabolic profile of normal and cancerous prostate cells has provided a window into the carcinogenesis process, highlighting the importance of cellular metabolism in this process.

Studies of prostate metabolism clearly demonstrate that a combined knowledge of metabolic processes, genomics, and proteomics is fundamental for a proper understanding of the cancer process.

An expanded metabolic repertoire supports the reprograming of glucose, lipid, hormone, amino acid, and glycoprotein metabolic pathways during malignant transformation and tumor development in both the prostate gland and cancer cells in general.

Some of these pathways, especially those involving glucose, lipids, and amino acids, have been extensively studied by metabolic imaging. Normal prostate glucose pathways are characterized by low oxygen consumption (4) and minimal oxidation of citrate, both suggestive of a typical tumor cell metabolic profile (5), but caused by a normal accumulation of zinc that inhibits m-aconitase activity (6). The observed metabolic transformation in PCa is an increase in citrate oxidation of malignant cells, which has led to the hypothesis of increased m-aconitase activity compared to the normal epithelial cells. However, as highlighted by Singh et al. (7), proteomic analyses has shown that the level of m-aconitase does not change in cancerous prostate tissue, although the elevated zinc level inhibits its activity in normal tissue. As a consequence, *in vivo* non-invasive metabolic imaging has shown a high level of citrate in normal prostate gland that significantly decreases in regions of cancer associated with a significant choline (Cho) increase (8).

In fact, metabolic pathways of lipids, and in particular phospholipids, are altered, as shown by *in vivo* 1H-MR spectroscopy imaging (MRSI) studies that detect elevated total Cho in regions of PCa and by *ex vivo* (9) and tissue (10) 1H-MR spectroscopy studies that detect the total Cho peak due to significant increases of free Cho, phosphocholine (PC), and glycerophosphocholine (GPC). The altered lipid pathway in PCa has also been studied using positron emission tomography (PET) imaging (11) with radiotracers, such as 11C-Cho (and 18F-Fluorocholine) and 11C-acetate.

The amino acid metabolic pathway has also been studied by PET, exploiting the fact that amino acid transport is upregulated in PCa cells.

The metabolic peculiarities of prostate gland metabolism have driven the development of the techniques for its study *in vivo*.

## *In Vivo* Techniques for Metabolic Imaging

Metabolic imaging refers to a set of molecular imaging methods that provide direct information about tissue metabolism. Molecular imaging may be defined as the visualization, characterization, and measurement of biologic processes at the molecular and cellular levels (12). Molecular imaging methods can detect specific biologic processes that change in cancer relative to surrounding normal tissue. Of course, it is desirable to have non-invasive instruments able to map the metabolism of various organs *in vivo*.

Many research methods are sensitive to tissue metabolism, and of these MRSI and PET are widely used in clinical practice and clinical research. Since the late 90s, both MRSI/MRI and PET/CT have been evaluated for PCa detection (8, 13, 14).

An MRSI exam is always associated with an MRI scan in order to have both metabolic and anatomical data. It is a non-invasive technique since non-ionizing radiation is used. Using MRSI, multiple metabolites can be spatially resolved in a single imaging protocol with a spatial resolution that can be reduced below 0.5 cm^3^; nevertheless with clinical systems, usually only a limited number of metabolites are present at concentrations that allow useful spatial mapping. This significantly limits the metabolic characterization of tumors. In prostate gland citrate, creatine, polyamine, and Cho-group resonances are detectable on 1.5- to 3-T scanners and the (choline + creatine)/citrate ratio is known to increase with tumor aggressiveness (15, 16). In a systematic review and meta-analysis of the use of combined MRI and MRSI in PCa pooled sensitivity for the evaluation of primary tumor was 68% (95% CI, 56–78%) with and pooled specificity 85% (95% CI, 78–90%). Subdividing patient into low and high risk subgroups, the sensitivity was lower in low-risk patients [58% (46–69%) vs. 74% (58–85%); *p* > 0.05] but higher for specificity [91% (86–94%) vs. 78% (70–84%); *p* < 0.01] (17).

Recently, developments in pulse sequences for MRSI have increased spectral resolution (18), and improved signal-to-noise (SNR) and volume selection (19).

Magnetic resonance spectroscopic imaging, unlike PET, detects static concentrations of endogenous molecules rather than tracer uptake. PET imaging is mostly used in association with a CT scan to sum up metabolic and anatomical information. A PET-CT scan involves exposure to ionizing radiation. PET is characterized by a high sensitivity in the detection of gamma radiation but with a spatial resolution which is not below a few millimeters. One of the drawbacks of PET for metabolic imaging is the absence of chemical information. The most commonly used tracer for PET imaging is [18F]-fluoro-2-deoxyglucose (FDG) but not all tumors consume glucose, so specific tracers have to be found for different tumors. The use of Cho radiotracers (11C-Cho and 18F-fluorocholine) and 11C-acetate is based on increased cellular membrane synthesis in PCa. Cho is overexpressed in cancer cells and is used for the synthesis of phosphatidylcholine, a prerequisite for cell membrane formation. Like Cho, acetate is a substrate needed for lipogenesis and hence important for increased cell membrane synthesis during PCa. Just as with FDG, uptake level of lipogenesis tracers in benign tissue and PCa can overlap, determining the low specificity of these tracers for primary PCa detection (20). In a systematic review and meta-analysis of 11C-acetate PET/CT in PCa, pooled sensitivity and pooled specificity for evaluation of primary tumor were 75.1% (95% CI: 69.8–79.8%) and 75.8% (95% CI: 72.4–78.9%), respectively (21). 11C-Cho showed a sensitivity of 66% and specificity of 81% for localization of primary PCa on a sextant histopathologic analysis (22). Although lipogenesis tracers present limitations for detection of primary PCa, they might be useful for a minority of newly diagnosed patients in whom distant metastatic disease is highly suspected on the basis of clinical data (serum PSA level >20 ng/ml, Gleason score 8–10, locally advanced tumor). In a systematic review, Evangelista et al. reported a sensitivity and a specificity of 19–90 and 88–98%, respectively, for detection of metastatic lymph nodes (2). These results are linked to a high false-positive rate due to inflammation and a high false-negative rate linked to micrometastatic lymph node disease undetectable by the majority of PET/CT scanners. More rigorous imaging trials are required to validate methods, and determine their sensitivity, to reduce the extreme variability of these results. The role of Cho PET/CT for restaging of biochemically recurrent PCa has been confirmed, particularly when the PSA level becomes elevated. One systematic review and meta-analysis including 19 selected studies with a total of 1555 patients revealed a pooled sensitivity of 85.6% and pooled specificity of 92.6% (23). In a recent study with a large cohort of patients, the accuracy of 11C-Cho PET/CT for the detection of sites of metastatic disease in PCa patients with biochemical relapse was confirmed. The authors also showed that in their series the PSA level was the main predictor of a positive scan with 1.16 ng/ml as the optimal cut-off value. They asserted that in the majority of positive scans they detected oligometastatic disease, potentially treatable with salvage therapies (3).

Other tracers have been and are currently being investigated (see Advanced Protocol and Perspectives) but only Cho and very recently 18F-FACBC have FDA approval for clinical use.

One of the latest molecular imaging modalities is hyperpolarized 13C-MRSI. It combines the advantages of MRSI with an enormous gain in sensitivity in 13C detection due to the method of hyperpolarizing the molecules.

The dynamic nuclear polarization technique has been applied to 13C-MRSI (24): it is based on the transfer of polarization from the electron spins of paramagnetic centers embedded in a glassy frozen solution to neighboring nuclear spins (i.e., 13C nuclei of an informative biomolecule) through dipolar interactions. The solid solution is then rapidly dissolved and prepared for an intravenous injection. MRSI data must be obtained as rapidly as possible after dissolution because the enhancement is lost with the spin–lattice relaxation of the biomolecule. With hyperpolarized 13C-MRSI, it is possible to monitor substrate uptake and the metabolism of endogenous biomolecules (25) by studying metabolic fluxes *in vivo*. Polarization methods, sequences, coils, and substrates (26, 27) have all been developed since 2007 with the aim of improving the accuracy of PCa evaluation.

## Current Use of Metabolic Imaging in Clinical Practice

**Figure 1:**
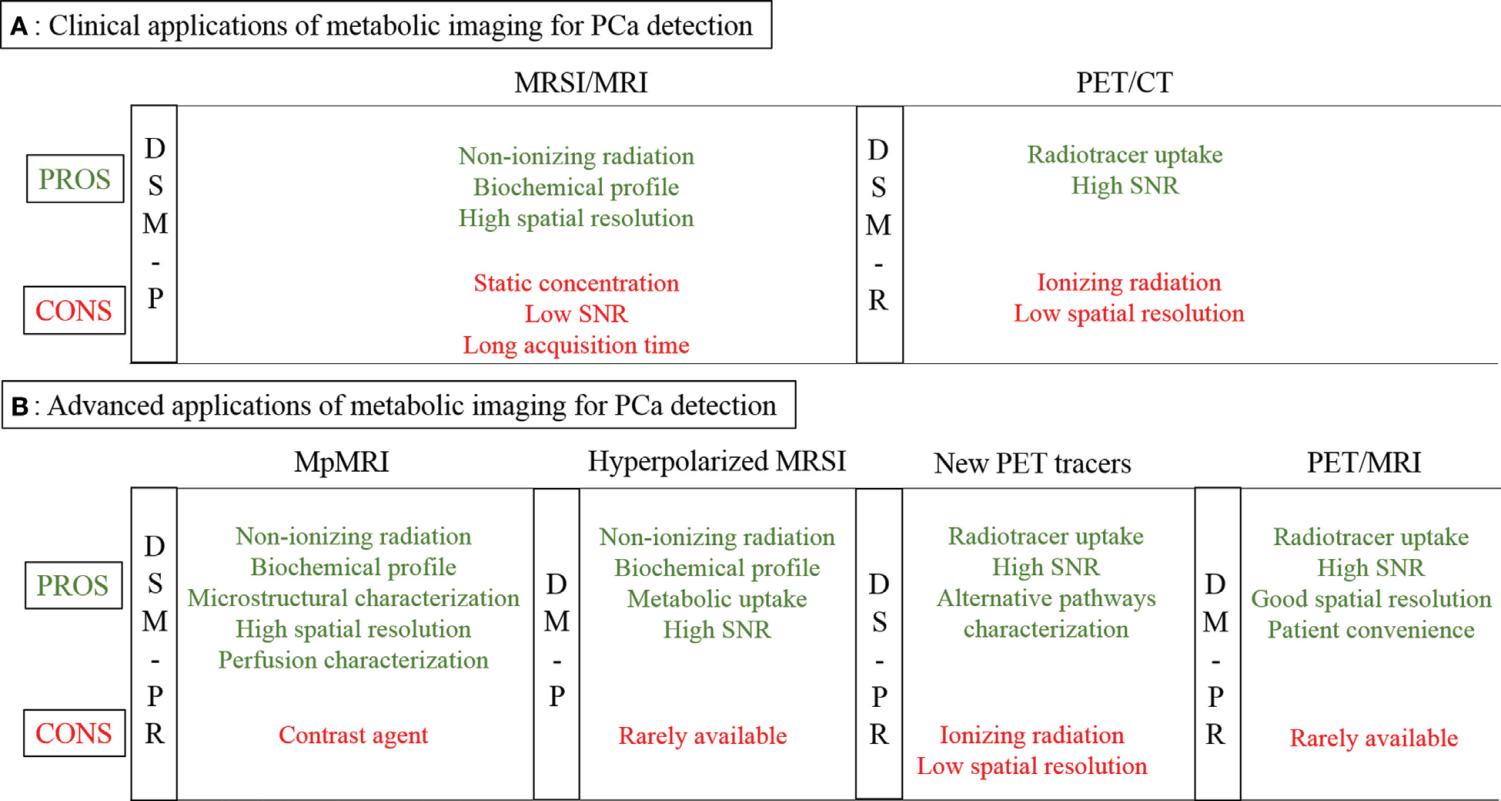
**Metabolic imaging in PCa detection**. **(A)** Clinical applications of MRSI/MRI and PET/CT (standard tracers): pros and cons. **(B)** Advanced applications: hyperpolarized MRSI, mpMRI, PET/CT (new tracers), and PET/MRI: pros and cons. Abbreviations: MRSI, magnetic resonance spectroscopic imaging; MRI, magnetic resonance imaging; PET, positron emission tomography; CT, computed tomography; mpMRI, multiparametric MRI; P, primary tumor; R, recurrence; D, diagnosis; S, staging; M, monitoring.

**Figure 2:**
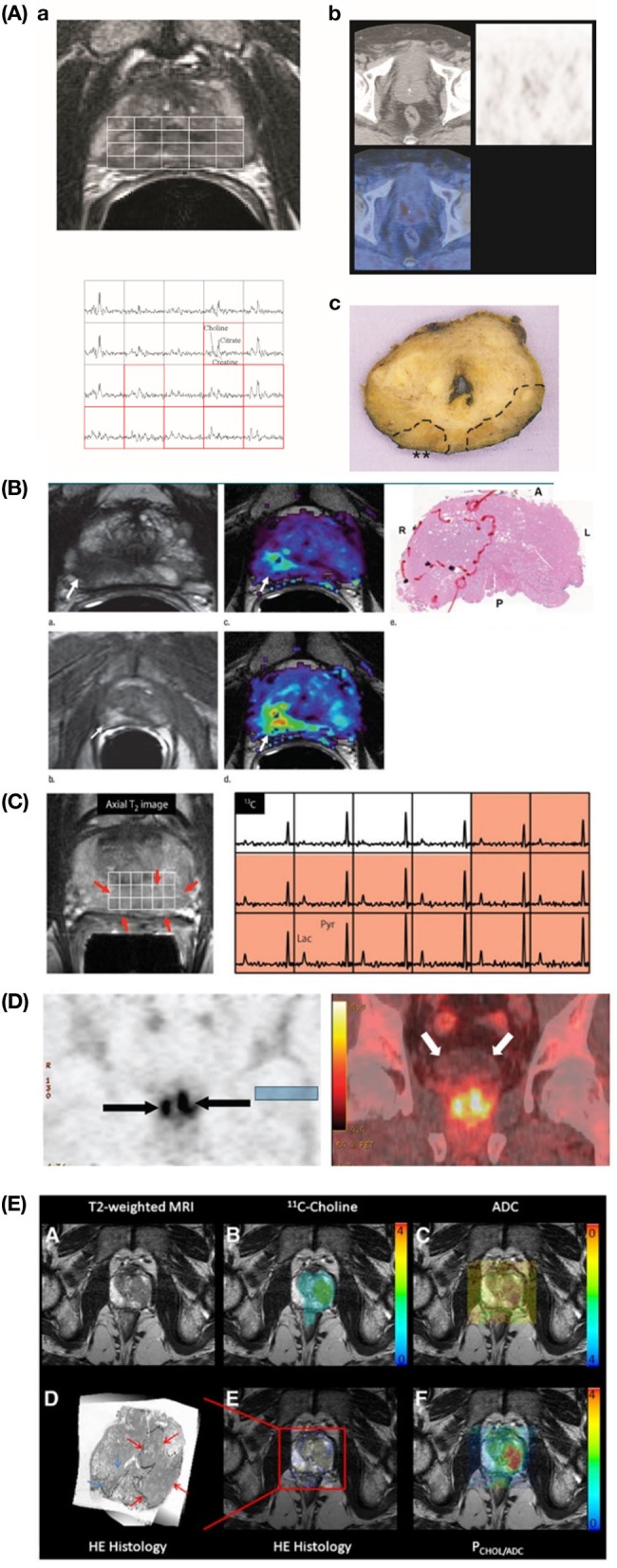
**Example images showing advanced metabolic imaging approaches to PCa detection**. **(A)** MRSI/MRI and PET/CT. Transverse T2-weighted MR image (*upper left*) shows bilateral signal hypointensities and corresponding 3D MR spectroscopic spectra (*lower left*) show bilateral abnormalities [mean (Choline + Creatine)/Citrate ratio = 0.95 on right side and 1.10 on left] indicative of cancer, while corresponding PET/CT transverse images *(upper right)* do not show any relevant pathologic focal accumulation of 11C-choline [background maximum SUV (standardized uptake value) = 2.5]. Corresponding pathologic specimen (hematoxylin–eosin stain; original magnification, ×1) (*lower right*) shows bilateral posterior adenocarcinoma (T3aNXMX, Gleason score 4 + 3) with right extracapsular extension (**). Reproduction with permission from Ref. (14) (RSNA). **(B)** mpMRI in a 62-year-old man with PCa. Axial T2-weighted MR image (*upper left*) demonstrates a low-signal intensity focus (arrow) at right apex mid peripheral zone suspicious for PCa. Raw dynamic contrast-enhanced MR image (*lower left*) and *k*trans (wash in) (*upper middle*) and *k*ep (wash out) (*lower middle*) maps help localize tumor (arrow). Histopathologic slide at apex mid prostate level (*upper right*) confirms presence of tumor (Gleason score, 8) more anteriorly (red line), secondary to distortion and shrinkage of specimen. A, anterior; L, left; P, posterior; R, right. Reproduction with permission from Ref. (28) (RSNA). **(C)** 13C-hyperpolarized MRSI in a patient, who had a serum PSA of 4.5 ng/ml, was originally diagnosed with bilateral biopsy-proven Gleason grade 3 + 3 PCa, and received the highest dose of hyperpolarized [1-13C]pyruvate (0.43 ml/kg). On the *left*, an axial T2-weighted images and on the *right* the corresponding spectral array with the area of putative tumor highlighted by pink shading. A region of tumor was observed on the T2-weighted images (red arrows). From Ref. (29) (Nelson SJ, Kurhanewicz J, Vigneron DB, Larson PE, Harzstark AL, Ferrone M, et al. Metabolic imaging of patients with prostate cancer using hyperpolarized [1-^13^C]pyruvate. *Sci Transl Med* (2013) 5(198):198ra108. Reprinted with permission from AAAS.) **(D)** Coronal PET (*left*) and CT fused (*right*) anti-18F-FACBC images of 63-year-old male patient with pathologically proven bilateral prostate carcinoma (arrows on the *left*). Note little bladder activity (white arrows on the *right*). This research was originally published in JNM (30). Schuster et al.© by the Society of Nuclear Medicine and Molecular Imaging, Inc. **(E)** PET/MRI fusion imaging in high-grade PCa. Specific image information derived from 11C-choline PET (*upper middle*), ADC (apparent diffusion coefficient) DWI (*upper right*), hematoxylin–eosin (HE) histology (*lower left*), and parametric fusion PET/MRI using PCHOL/ADC* (*lower right*) is coregistered with transaxial T2-weighted MRI (*upper right*). Color bars indicate 11C-choline SUV (standardized uptake value) (*upper middle*), PCHOL/ADC* (*lower right*), and inverted ADC (*upper right*). Zoomed registered HE histology slice is shown for increased clarity (*lower left*). At histology, Gleason 4 + 3 lesion is located in left lobe of prostate (red arrows) in peripheral and central zone, which is identified on registered imaging, whereas additional low-volume Gleason 3 + 3 lesion in right lobe is not identified (blue arrows) $\text{*}\mathrm{PCHOL/ADC}=\frac{11C-\mathrm{Choline}\;\mathrm{SUV}}{\mathrm{ADC}\;+\;0.001}$. This research was originally published in JNM. Park et al. (31) © by the Society of Nuclear Medicine and Molecular Imaging, Inc.

Figure 1A outlines the most common methods used for metabolic imaging in clinical practice. Their main technical characteristics are highlighted, divided into pros and cons. The utility of each technique for diagnosis, staging, and/or monitoring of therapy is indicated in vertical columns, subdivided into primary detection or recurrence. Figure 2A shows example images for these techniques.

Although an extensive literature shows how much these non-invasive imaging techniques are used to assess PCa in Europe, guidelines for PCa staging still indicate MRSI/MRI and PET/CT (32) as being under evaluation. The accuracy of MRSI/MRI aids in tumor localization within the peripheral zone, increasing the accuracy of extracapsular extension detection among less-experienced readers, and decreasing inter-observer variability. Nevertheless, given difficulties in interpreting signal changes related to post-biopsy hemorrhage and inflammatory changes of the prostate, and the unquantifiable but significant inter- and intra-observer variability seen between both non-dedicated and dedicated radiologists, that may lead to under- or overestimation of tumor presence and the local extent of disease, this technique is appropriate only for a limited number of cases. The overall accuracy of 11C-Cho PET in defining local tumor stage has been reported to be around 70% (33). PET tends to understage PCa, and has a limited value for making treatment decisions in patients with clinically localized PCa. Thus, routine clinical use of 11C-Cho PET cannot be recommended for detecting and staging primary PCa. By contrast, 11C-Cho PET/CT is regarded as a well investigated modality for the restaging patients presenting with elevated PSA levels following radical treatment of PCa (34).

The ACRIN study on PCa localization by MRI and MRSI (35) showed that the accuracy of combined 1.5-T endorectal MR imaging–MR spectroscopic imaging for sextant localization of peripheral zone PCa is equal to that of MR imaging alone. Nevertheless, many publications have showed that the combination of MRI and MRSI even in selected applications (such as diagnosis of cancer in men with more than one previous negative biopsy) really improves MRI performance (8, 21, 36, 37). This disagreement with the ACRIN study is explained by the expertise of radiological staff (together with physicists, chemists, and biologists) in acquisition of MR spectra, in post-processing and by the close collaboration with urologists in approaching the diagnosis of each single patient. To overcome this methodological problem, fully automated procedures to assess MR spectra have been proposed; the classification capabilities of automated pattern recognition approaches are equal to or better than manual methods (38, 39).

With regard to PET, its diagnostic power lies in its ability to stage disease, monitor treatment response, and detect recurrence, rather than in initial diagnosis (40); hence, PET is still prescribed only for equivocal cases and not for clinical routine.

It remains an open question whether, in the light of the existing literature and current diagnostic guidelines, MRI/MRSI and PET are needed to increase accuracy in PCa detection in clinical practice.

## Advanced Protocols and Perspectives

Figure 1B outlines the most recent advanced approaches for metabolic imaging. As for Figure 1A, their main technical characteristics are highlighted, dividing between pros and cons. The utility of each technique for diagnosis, staging and/or monitoring of therapy is indicated in vertical columns. Figure 2 shows example images of these advanced metabolic imaging approaches.

Multiparametric MRI (mpMRI) refers to the use of complementary MR techniques to improve the detection, characterization and staging of PCa by T2-weighted MRI, MRSI, DTI, and dynamic contrast-enhanced MRI scans in the same session. The combination of such acquisitions gives anatomical, microstructural, functional, and metabolic information with the high spatial resolution of MRI. MpMRI data appears to be the most accurate imaging method for localizing primary PCa and staging primary or recurrent PCa (28, 41, 42). An mpMRI acquisition has no particular technical limitation apart from the use of a contrast agent for DCE imaging. Nevertheless, the challenge to the diffusion of mpMRI is the need for specialized clinical staff and the need to present data to clinical colleagues in a simple but meaningful way in spite of its complexity. Shared acquisition, post-processing, and interpretative protocols become essential. This requirement has inspired the definition of the PI-RAD score, specifically formalized for predicting the presence of cancer by mpMRI findings from of T2w, T1w, DWI, and DCE acquisitions (43). The use of this score provides a combined evaluation of all functional and structural MR imaging scans; nevertheless a quantitative score for all techniques is still lacking. On the contrary, a quantitative evaluation for MRSI, which could be part of mpMRI protocol but is absent from the PI-RAD scoring system, has already been evaluated (44) and applied (14, 45).

Magnetic resonance spectroscopic imaging of hyperpolarized nuclei is a relatively new and sophisticated approach, which offers the possibility of monitoring tumor metabolism by the injection of labeled substrates and observation of their metabolic products without the use of ionizing radiation. Metabolic imaging with hyperpolarized 13C pyruvate, which provides information about tissue biochemistry, has been demonstrated to greatly increase the sensitivity of MRI in detecting PCa (46). Nevertheless, hyperpolarization is a highly demanding procedure due to both the process that drives nuclei to a non-stable but more sensitive condition and the rate of the exponential decay process, which limits acquisition time to a few tens of seconds. Thus, a number of hurdles must be overcome to transfer these methods to a clinical setting; the major one are technical, and regard the MR scanner and the polarizer, but there is the important matter of the safety of the substrate: to date no significant adverse effect have been observed in tolerability studies performed in test animals and the first application in human patients has shown no dose limit toxicity (29).

In PET imaging, several promising radiotracers are currently being investigated for the evaluation of PCa but not based on the detection of glucose or fatty acid metabolism since glucose and lipogenesis tracers have demonstrated limitations for detection of primary PCa. These new radiotracers aim to monitor amino acid metabolism (anti-18F-FACBC), DNA synthesis, and the expression and activity levels of a variety of receptors, enzymes, and other cancer-specific and non-specific biomarkers.

The radiotracer 1-amino-3-florine-18-florocyclobutane-1-carboxylic acid (18-FACBC) exploits the fact that amino acid transport is upregulated in PCa cells. The low urinary excretion of this radiotracer also allows 18-FACBC uptake in malignant tumor cells to be detected more accurately. Few papers have been published on the role of 18F-FACBC for the evaluation of primary or recurrent PCa. Schuster et al. compared the diagnostic performance of 18F-FACBC and 111In-capromab in 93 patients reporting a higher accuracy for 18F-FACBC and an upstaging of recurrent disease in at least 25% of patients (30). Nanni et al. recently confirmed the higher accuracy of 18F-FACBC compared to 11C-Cho in restaging of PCa, particularly in patients with low PSA levels (<1 ng/ml) (47).

To date, about 1000 patients have been studied with 68Ga-PSMA (prostate-specific membrane antigen) in different clinical indications, such as initial staging and restaging of disease and in comparison with other common radiopharmaceutical agents for PET imaging, such as radiolabeled Cho. PSMA is a membrane glycoprotein with an extensive extracellular domain, a transmembrane segment, and an intracellular domain. PSMA is normally expressed in epithelial cells within the prostate and is strongly upregulated at all stages of PCa. An increase in PSMA expression has been associated with tumor aggressiveness, metastasis, and disease recurrence, providing a rational target for ligand–receptor-based imaging and therapy. Two groups have demonstrated that 68Ga-PSMA PET/CT is superior to 18F-Cho PET/CT and conventional imaging modalities (48, 49). Eiber et al. evaluated the accuracy of 68Ga-PSMA PET/CT in 248 patients with biochemical relapse and showed a patient-based detection rate of 89.5% with a detection rates for PSA levels of ≥2, 1 to <2, 0.5 to <1, and 0.2 to <0.5 ng/ml were 96.8, 93.0, 72.7, and 57.9%, respectively (50). Clinically, it is very important that 68Ga-PSMA PET/CT has a high detection rate for low values of PSA allowing the site of recurrence to be detected within the windows of curability. Further studies are required to confirm these data.

Finally, the role of PET/MR has not yet been established: it offers the possibility of enhancing patient convenience by providing a single metabolic imaging session to replace separate MRI and PET/CT scans, reducing patient anxiety, total scan time, and recalls for repeated scanning. Although preliminary results are encouraging (31, 51, 52), it is unknown whether this sophisticated modality will demonstrate the high performance of MRI in the staging of the primary cancer and the metabolic profiling of lesions offered by 11C-Cho PET. It would be interesting to evaluate the inclusion of MRSI in PET/MR protocols.

In conclusion, the challenge for researchers is to improve the accuracy of metabolic imaging techniques for the diagnosis of PCa, and in consequence to allow improvements in therapy. MpMRI, 13C hyerpolarization for *in vivo* 13C-MRI, new tracers for PET scan, and the combination of PET with MRI are all new metabolic imaging tools that show promise in meeting this challenge.

However, all the cited advanced metabolic techniques have to be evaluated considering both their potential capabilities and their clinical applicability. The most promising technique can then be integrated in multiparametric protocols that must be standardized if they are to be applied in a clinical context.

## Author Contributions

CT: literature research, manuscript draft, and manuscript revision. CP: literature research and manuscript draft. DNM: manuscript revision and English revision. RS: literature research and manuscript revision. RL: literature research and manuscript revision.

## Conflict of Interest Statement

The authors declare that the research was conducted in the absence of any commercial or financial relationships that could be construed as a potential conflict of interest.
